# Physicochemical Index Analyses of the Egg White in Blue-Shelled Eggs and Commercial Brown-Shelled Eggs during Storage

**DOI:** 10.3390/foods12244441

**Published:** 2023-12-11

**Authors:** Huanhuan Wang, Ying Ge, Yinghui Wei, Qinghai Li, Xuedong Zhang, Jinghui Fan

**Affiliations:** Hangzhou Academy of Agricultural Sciences, Hangzhou 310024, China; geying1122@126.com (Y.G.); wyh2017105016@njau.edu.cn (Y.W.); lflou@hz.cn (Q.L.); bigzhengliang@hotmail.com (X.Z.); ufanta@163.com (J.F.)

**Keywords:** egg, brown eggshell layer, blue eggshell chicken, albumin protein, S-ovalbumin, ovomucin, lysozyme

## Abstract

To compare the physical and chemical changes in egg whites during storage, assisting in the evaluation of differences in egg freshness between various chicken breeds, we chose 240 blue-shelled eggs (Blue group) and 240 commercial brown-shelled eggs (Brown group) that 28-week-old hens had laid. In this study, all eggs were stored at 25 °C. The egg weight, egg components’ weight and proportion, Haugh Unit value and the contents of S-ovalbumin, ovomucin and lysozyme in the thick albumen (KA) and thin albumen (NA) were measured at eight time points every 3 days until the 21st day of storage. The eggshell, yolk and KA proportions in the Brown group were significantly lower, whereas the NA proportion was significantly higher than that in the Blue group (*p* < 0.001). The Haugh Unit value and S-ovalbumin in the Brown group were significantly higher, whereas KA ovomucin and NA lysozyme were significantly lower than those in the Blue group (*p* < 0.001). There existed significant negative correlations between the KA and NA, irrespective of weight or proportion. The Haugh Unit value was significantly positively correlated with lysozyme and ovomucin, but significantly negatively correlated with S-ovalbumin. During storage, the KA weight (proportion), Haugh Unit value, lysozyme and ovomucin decreased, whereas the NA weight (proportion) and S-ovalbumin increased. At each time point, the NA lysozyme in the Brown group was lower than that in the Blue group (*p* < 0.05). After storage for 6 days, the KA ovomucin in the Brown group began to be lower than that in the Blue group (*p* < 0.05). The study showed that the weight (proportion) differences in egg components between blue-shelled eggs and commercial brown-shelled eggs are mainly due to the NA. The Haugh Unit value and albumin protein indexes of blue-shelled eggs were better than those of brown-shelled eggs, and showed mild changes during storage, indicating the better storage performance of blue-shelled eggs.

## 1. Introduction

Eggs are rich in nutrients such as protein, lipids and vitamins, making they one of the most important food sources for humans, and often being consumed at freshness or after storage [1]. From the perspective of organization, eggs consist of three major structures: eggshell, egg yolk and egg white (i.e., albumen). Among them, the egg white accounts for the highest proportion, usually around 60% [2]. Because of their excellent beating, emulsifying and gelling characteristics, egg whites are widely used in various food processes [3]. It has been found that the nutritional components of egg whites are mainly albumin, ovomucin and lysozyme [4], and the compounds formed via the interaction of these albumen proteins are important factors that maintain the viscosity and elasticity of egg whites [5]. During storage, egg quality indexes such as physical properties, chemical compositions and nutritional and hygienic indexes change over time. Especially in egg whites, the changes are significant and crucial, e.g., it was reported that the Haugh Unit value gradually decreased, whereas the content of S-ovalbumin gradually increased [6,7,8,9]. However, as an important indicator of egg freshness, the Haugh Unit value is calculated only using the measured height of the thick albumen (KA), a thick part of egg white, ignoring the thin albumen (NA) [10]. Research on the physicochemical changes in the two parts of the egg white during storage is still lacking.

Equally, some studies have found that there were differences in the levels and changes in the Haugh Unit values and lysozyme between eggs from different chicken breeds [11,12]. Generally, commercial layers, e.g., Hy-Line brown and white eggshell layers and ISA brown and white eggshell layers, have high levels of egg production after long-term selection of breeding, whereas traditional (i.e., indigenous, native or local) chickens exhibit lower egg-laying rates and lower egg weights [13,14]. On the consumer market, commercial eggs laid by commercial layers absolutely occupy the major proportion; meanwhile, the eggs laid by indigenous chickens, e.g., blue-shelled eggs, are also welcomed in some countries and districts [15]. However, there is still a need for additional research comparing the egg qualities of these two types of eggs.

Thus, this study undertook the measurement of egg quality indexes during the storage of blue-shelled eggs and commercial brown-shelled eggs, with a focus on some special protein analyses of the KA and NA, aiming to provide scientific proof for revealing the changes in different eggs during storage and helping the comparison and evaluation of egg freshness.

## 2. Materials and Methods

### 2.1. Samples and Groups

The blue-shelled eggs for testing (referred to as the Blue group) were collected from a chicken line at Hangzhou Academy of Agricultural Sciences, originating from the segregating progeny of a cross between two Chinese indigenous breeds, Dongxiang blue eggshell chicken and Jiangshan black-bone chicken. The brown-shelled eggs (referred to as the Brown group) were from a population of commercial Hy-Line brown eggshell layers. The eggs in fresh form from these two groups are shown in Figure S1. The hens of the two groups were both raised in the same environment and fed the same diet. The major nutrients during their laying periods were 17.5% crude protein and 2850 kcal/kg metabolism energy. At 28 weeks of age, 240 fresh eggs from each group were stored in an incubator (HWS-160, Ningbo Jiangnan Instrument Factory, Ningbo, China) under constant temperature and humidity (25 °C and 65%, respectively). At eight time points during their storage (i.e., day 0, day 3, day 6, day 9, day 12, day 15, day 18 and day 21), 30 randomly chosen eggs from each group were analyzed for their physicochemical indexes, which are described below.

### 2.2. The Measurement of Eggs’ Main Components

After being weighed, 15 eggs from each group were broken and separated into the eggshell and inner content. Using a yolk separator, the inner content was separated into egg yolk and egg white, and then the egg white was further separated into the KA and NA using a 40-mesh sieve. The weights of the eggshell, yolk and NA were measured, and the weight of the KA was obtained from the egg weight (EW) minus the weights of the above three components. Their corresponding proportions were calculated by dividing their weights by the EW.

### 2.3. Haugh Unit Value Measurement

The other 15 eggs from each group were also weighed and broken, and then their inner contents were tiled on horizontal glass plates. Using a vernier caliper, the albumen heights at three points of an equilateral triangle were measured and averaged; then, the Haugh Unit value was calculated using Equation (1):

Haugh Unit = 100 × Log (albumen height − 1.7 × EW^0.37^ + 7.57). (1)

### 2.4. S-ovalbumin, Lysozyme and Ovomucin Detection

The KA and NA of six randomly chosen eggs from each group were sucked to detect the S-ovalbumin, ovomucin and lysozyme. Referencing the method of Huang et al. [16], 2 g KA (or NA) was put into 10 mL of phosphate buffer (pH = 7.5), and magnetically stirred for 5 min. Then, two 2.5 mL solutions were put into two tubes, one at room temperature (i.e., unheated) and the other in a water bath at 75 °C for 30 min (i.e., heated). Afterward, 5 mL precipitant, a solution of 0.5 mol/L sodium chloride mixed with 0.1 mol/L sodium acetate, was added to each tube and mixed well, then stewed for 10 min and centrifuged at 12,000× *g* for 5 min. Then, 0.5 mL of supernatant fluid was mixed with 1 mL of biuret solution, stewed for 30 min and the absorbance was measured at 540 nm using a microplate reader (Tecan Infinite M200 PRO, Männedorf, Switzerland). The content of S-ovalbumin in the KA (or NA) was calculated using Equation (2):

S-ovalbumin = 100 × (OD_540 (heated)_/OD_540 (unheated)_). (2)

Referencing the method of Liu et al., 75 mg of lysozyme standard (Sigma-Aldrich L4919, Taufkirchen, Germany) was placed into a 50 mL volumetric flask with 0.9% sodium chloride, and serially diluted to form lysozyme standard solutions at concentrations of 100, 80, 60, 50, 40, 30, 20 and 10 µg/mL. The absorbances of the solutions were measured at 281 nm using a microplate reader, and the corresponding standard curve was formed with its regression equation. Further, 1 g of the KA (or NA) of each egg was diluted 50 times with 0.9% sodium chloride, the pH regulated to 4.5 using acetic acid, water-bathed at 77 °C for 10 min and stewed for 1 h. After centrifuging at 4500× *g* for 15 min, the biuret solution was sucked and its absorbance was measured at 281 nm; then, its lysozyme content was calculated using the standard curve equation. The content of lysozyme in the KA (or NA) was obtained by multiplying the lysozyme concentration of the solution by the dilution factor of the solution.

Referencing the method of Omana et al. [17], 10 g KA (or NA) of each egg was added to three times the volume of 0.1 mol/L sodium chloride and stirred for 30 min at 4 °C. After regulating the pH to 6 using 2 mol/L hydrochloric acid, the solution was stewed overnight at 4 °C, then centrifuged for 10 min at 11,000× *g* using a high-speed freezing centrifuge (Hitachi CR22GII, Shizuoka, Japan). The sediment was resuspended with 0.5 mol/L sodium chloride, the pH regulated to 6, then stirred for 30 min at 4 °C and stewed overnight again. The solution was recentrifuged for 10 min at 11,000× *g* at 4 °C; then, the sediment was freeze-dried using a vacuum-freezing drying oven (Labconco 7400030, Kansas, MO, USA) and weighed. The content of ovomucin in the KA (or NA) was calculated using Equation (3):

Ovomucin = 1000 × (sediment weight/10)(3)

### 2.5. Statistics and Analyses

The mean and standard error of each index were summarized using the JMP Pro 14 software (SAS Institute Inc., Cary, NC, USA), and group and storage were both categorical variables. Meanwhile, using Student’s *t*-test in one-way analysis of variance, the difference in index was determined, at significance levels of *p* < 0.05, *p* < 0.01 and *p* < 0.001.

## 3. Results

### 3.1. The Means and Effect Situation of Eggs’ Indexes

Table 1 shows that the EW and weights of the main components (i.e., eggshell, yolk, KA and NA) in the Brown group were all significantly higher than those in the Blue group (*p* < 0.001). The proportions of eggshell, yolk and the KA in the Brown group were all significantly lower than those in the Blue group (*p* < 0.001), whereas the proportion of the NA was significantly higher (Brown vs. Blue, *p* < 0.001). Among the physicochemical indexes, the Haugh Unit value and S-ovalbumin content in the Brown group were significantly higher than those in the Blue group (*p* < 0.001), whereas the lysozyme of the NA and ovomucin of the KA were significantly lower (Brown vs. Blue, *p* < 0.001). The results of the effect analyses showed that group (i.e., breed) and storage were both important factors causing significant differences in most measured indexes of the eggs. Moreover, the cooperative effects of group and storage on the Haugh Unit value and NA proportion were significant (*p* < 0.001).

### 3.2. The Correlations between the Main Components of Egg

Table 2 shows that the correlations between EW and the weight (and proportion) of the KA were all significantly positive, in both the Brown and Blue groups, whereas the correlations between EW and the proportion of the NA were significantly negative. Also, in both groups, the correlations between the weight and proportion of the eggshell, yolk, KA and NA were all significantly positive. The correlations between the weight (and proportion) of the yolk and that of the KA were significantly negative, whereas the correlations between the yolk and NA were positive. However, the correlations between the eggshell and other indexes in the Brown group were reversed in the Blue group.

Table 3 shows that the correlations between the Haugh Unit value and lysozyme (or ovomucin) content were all significantly positive in both the Brown and Blue groups, whereas the correlations between the Haugh Unit value and S-ovalbumin were significantly negative. Also, in both groups, the correlations between the S-ovalbumin and lysozyme (or ovomucin) content were all significantly negative, whereas the correlations between the lysozyme and ovomucin content were significantly positive.

**Figure 1:**
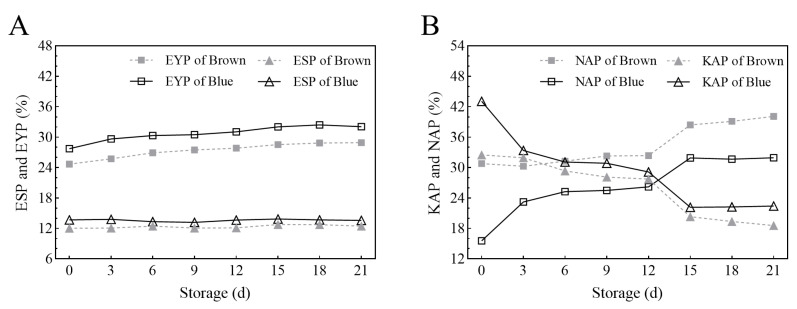
Changes in the proportion of the main components of egg during storage. Subfigure (**A**) shows the changes in eggshell proportion and yolk proportion, whereas subfigure (**B**) shows the changes in the thick albumen proportion and the thin albumen proportion. Brown: the Brown group of eggs from commercial brown eggshell layer; Blue: the Blue group of eggs from blue eggshell chicken; ESP: eggshell proportion; EYP: egg yolk proportion; KAP: thick albumen proportion; NAP: thin albumen proportion.

Figure 1 only shows the changes in proportions of the eggs’ main components because their weight changes were similar. Figure 1A shows that the eggshell proportion remained constant during the whole storage, whereas the yolk proportion increased at a degree of four percent. Figure 1B shows that the decreasing degree of KA proportion in the Brown group was lower than that in the Blue group (14% vs. 21%), whereas the increasing degree of NA proportion in the Brown group was lower than that in the Blue group (9% vs. 16%). The KA proportion in the Blue group was higher and the NA proportion was lower than that in the Brown group at each time point of storage. Before 6 d, the decrease in the KA proportion and increase in the NA proportion in the Blue group was more obvious than those in the Brown group. In the period from 12 d to 15 d, there also existed an obvious decrease in the KA proportion and an increase in the NA proportion in both groups.

### 3.3. The Changes in Egg White Indexes during Storage

**Figure 2:**
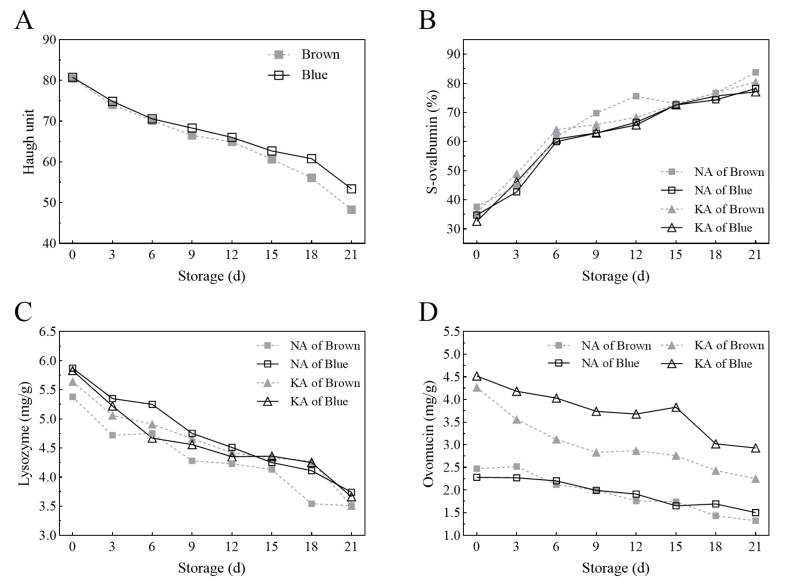
Changes in Haugh Unit and albumin protein components during storage. Subfigure (**A**) shows the changes in Haugh Unit, whereas subfigures (**B**–**D**) show the changes in S-ovalbumin, lysozyme and ovomucin in thick albumen and thin albumen, respectively. Brown: the Brown group of eggs from commercial brown eggshell layer; Blue: the Blue group of eggs from blue eggshell chicken; KA: thick albumen; NA: thin albumen.

Figure 2A shows that the Haugh Unit value of both groups decreased during storage, and the decreasing degree in the Brown group was higher than that in the Blue group. After 9 d, the difference became significant; then, after 15 d, the difference became highly significant. Figure 2B–D show that the S-ovalbumin content increased and the lysozyme and ovomucin content decreased in both groups during storage. The S-ovalbumin content in the Brown group was higher than that in the Blue group, and the increasing degree at early storage was significant (6 d vs. 0 d). The lysozyme content in the Brown group was mostly lower than that in the Blue group, and its difference in the NA was significant (Brown vs. Blue). After 3 d, the lysozyme content became significantly lower than that at 0 d. In addition, the ovomucin in the Brown group was mostly lower than that in the Blue group, and its content in the KA was significantly higher than that in the NA, at each time point of storage. During storage, the decreasing degree of ovomucin in the KA was more obvious than that in the NA, and after 6 d, the difference became significant (Brown vs. Blue).

## 4. Discussion

### 4.1. The Changes in Eggs’ Indexes during Storage

About 93% to 98% of the composition of eggshell is calcium carbonate, a very stable chemical under natural conditions and hardly affected by storage, which explains the result of this study that the eggshell proportion remained at about 13% during storage [18,19]. The yolk is rich in lipids, proteins and vitamins [20], and You et al. have reported that the size of the yolk increased and the elasticity of the membrane decreased during storage, following the increase in yolk weight and proportion [21]. This study also obtained a similar result.

In general, the egg white becomes thinner during storage, reflected in multiple indexes (e.g., Haugh Unit value, ovalbumin and ovomucin), which correlate with each other [22,23,24]. Scott and Silversides found that the KA height of the ISA commercial layer decreased from 9.16 mm at 0 d to 4.75 mm at 10 d during storage [25]. Akyurek and Okur found that the Haugh Unit value decreased from 91.48 to 52.11 after 14 days storage at 20 °C [26]. As shown in this study, the Haugh Unit value decreased from over 80 to below 60 after storage for 15 days. It has been reported that the thinning of egg whites during storage is caused by the conformational change from natural ovalbumin to S-ovalbumin, the structural damage of gel due to ovomucin degradation and lysozyme denaturation and its interaction with ovomucin [27,28,29]. The results of this study, that the S-ovalbumin in the KA and NA increases whereas the lysozyme and ovomucin decrease, is in accord with the above reports about the thinning mechanism of egg whites. The special finding of this study is that the ovomucin in the KA is significantly higher than that in the NA, but for S-ovalbumin and lysozyme, the differences between the KA and NA are not significant. Huang et al. and Fu et al. found the correlations between the Haugh Unit value and S-ovalbumin were −0.913 and −0.975, respectively [8,16]. Wang et al. found the correlation between the Haugh Unit value and ovomucin was 0.989 in Hy-Line brown-shelled eggs stored at room temperature [22]. In this study, the correlations between S-ovalbumin, ovomucin, lysozyme and the Haugh Unit value range from −0.96 to −0.92, from 0.93 to 0.96 and from 0.96 to 0.99, respectively, indicating the high correlations between the physicochemical indexes of egg whites.

### 4.2. The Differences in Egg Indexes between Various Chicken Breeds

Even in the same population of chickens, the egg quality, and especially the levels of albumen, are different because of the differences in the age of hens, the nutrients in theirdiet, diseases and the housing conditions [30]. For example, Kim et al., Nowaczewski et al. and Biesiasa-Drzazga et al. all reported that eggs from younger Hy-Line brown eggshell hens had a better quality of albumen than those from older hens [31,32,33]. However, the chickens in this study were the same age, housed under the same environment and fed the same diet. Thus, the genetic background (i.e., chicken breeds) became the main factor influencing the egg quality indexes. Moula et al. measured eggs from Belgian local breeds and commercial lines of chickens, and found that the proportion of albumen and Haugh Unit values in ISA brown eggshell layers were 62.0% and 79.0, respectively. Those values were higher and lower than those in the indigenous breeds, respectively [34]. Sokołowicz et al. compared different chicken breeds under organic rearing and found that the proportion of albumen and the Haugh Unit value in eggs from Hy-Line brown-eggshell layer were 60.77% and 89.94, respectively. Those values were both higher than those from blue–green eggshell Araucana chickens [35]. Lordelo et al. reported that the commercial hens produced heavier eggs with higher proportions of albumen and lower Haugh Unit values than four Portuguese breeds of chickens [36]. Hejdysz et al. studied eggs belonging to 14 different breeds or lines of hens worldwide. The results showed that Hy-Line brown eggshell layers had the highest proportion of albumen, and the lysozyme content in the albumen was lower than most other breeds [37]. In this study, the proportion of albumen of the Brown group reached 60.28% (25.95% in the KA plus 34.33% in the NA), which was significantly higher than that of the Blue group. This finding was similar to those of previous studies. But the results of the Haugh Unit value comparisons in the above studies were not consistent.

Considering the interactions between genetic factors and storage length, Scott and Silversides compared the egg quality of ISA brown eggshell and ISA white eggshell layers, and found that brown-shelled eggs had a higher proportion of albumen and a lower Haugh Unit value after storage for 10 days at room temperature [25]. Monira et al. measured the eggs from four commercial layers and found that White Rock had the highest Haugh Unit value, followed by White Leghorn and Barred Rock; the Haugh Unit value of Roland Red was the lowest after storage for 21 days [38]. Pu et al. compared the eggs from blue, white and tint eggshell chicken lines, and found that the decrease of Haugh Unit value was greatest for white-, then in order with tint- and then blue-shelled eggs after storage for 25 days at room temperature [39]. In this study, it was observed that the blue-shelled eggs had a reversed situation of KA and NA weight (and proportion) compared with the commercial brown-shelled eggs, i.e., the weight (and proportion) of the KA from a blue-shelled egg was higher than that of the NA, whereas the weight (and proportion) of the KA from a brown-shelled egg was lower than that of the NA. These results indicated that the difference between blue- and brown-shelled eggs was mainly in the NA. Along with the storage, the changes in physicochemical indexes between blue- and brown-shelled eggs also had a few differences. The decrease of Haugh Unit value and the increase of S-ovalbumin in the brown-shelled eggs were greater than those in the blue-shelled eggs. In particular, the brown-shelled eggs had a significantly lower level of ovomucin in KA and lysozyme in the NA than the blue-shelled eggs.

## 5. Conclusions

Compared with Hy-Line commercial brown eggshell layers, blue eggshell chickens had a significantly lower weight of egg compositions, but the components’ proportions were higher except for the NA, indicating that the weight (proportion) differences between the two breeds were mainly due to the NA. With lengthening storage, the Haugh Unit value and lysozyme and ovomucin levels decreased, whereas the S-ovalbumin level increased. The blue-shelled eggs had higher Haugh Unit values and lysozyme and ovomucin levels than brown-shelled eggs, whereas the S-ovalbumin was lower. Equally, the changes in most of the physicochemical indexes of the egg whites in the blue-shelled eggs were lower than those in the brown-shelled eggs during the whole storage, indicating the better storage performance of the blue-shelled eggs. These results are useful for revealing the mechanisms driving changes in egg quality.

## Figures and Tables

**Table 1:** The means, standard error and the significance of physicochemical indexes of eggs.

Index	Group		Effect Factor			Standard Error
	Brown	Blue	Group	Storage	Group * Storage	
Egg weight, g	56.51	46.89	***	**	NS	0.356
Eggshell weight, g	6.96	6.38	***	NS	NS	0.040
Yolk weight, g	15.44	14.36	***	***	NS	0.095
KA weight, g	14.77	13.84	*	***	NS	0.293
NA weight, g	19.34	12.32	***	***	**	0.299
Eggshell proportion, %	12.34	13.60	***	NS	NS	0.076
Yolk proportion, %	27.39	30.70	***	***	NS	0.229
KA proportion, %	25.95	29.32	***	***	*	0.535
NA proportion, %	34.33	26.38	***	***	***	0.478
Haugh Unit	65.15	67.29	***	***	***	0.584
KA S-ovalbumin, %	64.13	62.04	**	***	NS	1.506
NA S-ovalbumin, %	65.32	61.91	***	***	*	1.567
KA lysozyme, mg/g	4.60	4.61	NS	***	NS	0.064
NA lysozyme, mg/g	4.32	4.73	***	***	*	0.070
KA ovomucin, mg/g	3.01	3.74	***	***	NS	0.084
NA ovomucin, mg/g	1.92	1.94	NS	***	NS	0.053

KA: thick albumen; NA: thin albumen. *, ** and *** represent the significance (i.e., *p*-value) being lower than 0.05, 0.01 and 0.001, respectively. “NS” means non-significant.

**Table 2:** The correlations between the weight (and proportion) of the main components of egg. The values in the upper triangle are from the Brown group, and those in the lower triangle are from the Blue group.

Index	EW	ESW	EYW	KAW	NAW	ESP	EYP	KAP	NAP
EW		0.194 *	0.019	0.648 ***	−0.100	−0.405 ***	−0.480 ***	0.542 ***	−0.418 ***
ESW	0.581 ***		0.257 **	−0.131	0.086	0.817 ***	0.133	−0.178	0.016
EYW	−0.104	−0.019		−0.441 ***	0.209 *	0.230 *	0.866 ***	−0.490 ***	0.187 *
KAW	0.577 ***	0.262 **	−0.536 ***		−0.745 ***	−0.504 ***	−0.713 ***	0.990 ***	−0.892 ***
NAW	−0.155	−0.116	0.253 **	−0.832 ***		0.141	0.240 **	−0.802 ***	0.945 ***
ESP	−0.009	0.807 ***	0.051	−0.094	−0.030		0.408 ***	−0.488 ***	0.263 **
EYP	−0.525 ***	−0.268 **	0.899 ***	−0.704 ***	0.276 **	0.050		−0.707 ***	0.379 ***
KAP	0.472 ***	0.192 *	−0.559 ***	0.991 ***	−0.874 ***	−0.105	−0.680 ***		−0.912 ***
NAP	−0.337 ***	−0.220 *	0.254 **	−0.899 ***	0.981 ***	−0.027	0.358 ***	−0.921 ***	

EW: egg weight; ESW: eggshell weight; EYW: egg yolk weight; KAW: thick albumen weight; NAW: thin albumen weight; ESP: eggshell proportion; EYP: egg yolk proportion; KAP: thick albumen proportion; NAP: thin albumen proportion. *, ** and *** represent the significance (i.e., *p*-value) being lower than 0.05, 0.01 and 0.001, respectively.

**Table 3:** The correlations between Haugh Unit and the levels of albumin protein components. The values in the upper triangle are from the Brown group, and those in the lower triangle are from the Blue group.

Index	Haugh Unit	KA Lysozyme	NA Lysozyme	KA S-ovalbumin	NA S-ovalbumin	KA Ovomucin	NA Ovomucin
Haugh Unit		0.982 ***	0.968 ***	−0.937 ***	−0.920 **	0.946 ***	0.959 ***
KA lysozyme	0.974 ***		0.942 ***	−0.932 ***	−0.929 ***	0.942 ***	0.926 ***
NA lysozyme	0.983 ***	0.951 ***		−0.932 ***	−0.913 **	0.959 ***	0.936 ***
KA S-ovalbumin	−0.949 ***	−0.966 ***	−0.949 ***		0.976 ***	−0.991 ***	−0.929 ***
NA S-ovalbumin	−0.956 ***	−0.969 ***	−0.955 ***	0.994 ***		−0.968 ***	−0.947 ***
KA ovomucin	0.936 ***	0.90 2 **	0.927 ***	−0.880 **	−0.879 **		0.920 **
NA ovomucin	0.952 ***	0.878 **	0.969 ***	−0.886 **	−0.909 **	0.872 **	

KA: thick albumen; NA: thin albumen. ** and *** represent the significance (i.e., *p*-value) being lower than 0.01 and 0.001, respectively.

## Data Availability

Data are contained within the article and Supplementary Material.

## Supplementary Materials

The following supporting information can be downloaded at: https://www.mdpi.com/article/10.3390/foods12244441/s1, Figure S1: The photos of eggs from the Brown group and Blue group.

## References

[1] Sparks N. (2006). The hen’s egg—Is its role in human nutrition changing?. World Poult. Sci. J..

[2] Kovacs-Nolan J., Phillips M., Mine Y. (2005). Advances in the value of eggs and egg components for human health. J. Agric. Food Chem..

[3] Dong X., Zhang Y.Q. (2021). An insight on egg white: From most common functional food to biomaterial application. J. Biomed. Mater. Res. B. Appl. Biomater..

[4] Abeyrathne E.D.N.S., Lee H.Y., Ahn D.U. (2013). Egg white proteins and their potential use in food processing or as nutraceutical and pharmaceutical agents—A review. Poult. Sci..

[5] Wang H.J. (2019). Interaction of Four Egg White Proteins and Their Effects on the Quality and Allergenicity of Egg White Proteins. Master’s Thesis.

[6] Huang Q. (2012). Study on Correlation between S-ovalbumin and Egg Freshness and the Purification and Characteristics of S-Ovalbumin. Ph.D. Thesis.

[7] Liu M.Y., Ren F.Z., Lian Z.H., Guo H.Y. (2015). Characteristic change of main proteins in egg-white at different storage conditions. J Food Saf. Qual..

[8] Fu D.D., Wang Q.H., Ma M.H., Xu F. (2018). Correlation analysis between egg freshness indexes and S-ovalbumin content during storage. Food Sci..

[9] Yimenu S.M., Koo J., Kim J.Y., Kim J.H., Kim B.S. (2018). Kinetic modeling impacts of relative humidity; storage temperature; and air flow velocity on various indices of hen egg freshness. Poult. Sci..

[10] Eisen E.J., Bohren B.B., Mckean H.E. (1962). The haugh unit as a measure of egg albumen quality. Poult. Sci..

[11] Javůrková V.G., Pokorná M., Mikšík I., Tůmová E. (2019). Concentration of egg white antimicrobial and immunomodulatory proteins is related to eggshell pigmentation across traditional chicken breed. Poult. Sci..

[12] Lewko L., Krawczyk J., Calik J. (2021). Effect of genotype and some shell quality traits on lysozyme content and activity in the albumen of eggs from hens under the biodiversity conservation program. Poult. Sci..

[13] Rizzi C., Chiericato G.M. (2005). Organic farming production. Effect of age on the productive yield and egg quality of hens of two commercial hybrid lines and two local breeds. Ital. J. Anim. Sci..

[14] Xie Y.N., Xie S.T., Chen M.J., Rao S.T., Tan J.J., Zhou Z.B. (2021). Comparative analysis of production performance, egg quality and nutrient composition of laying hens of different breeds. J. Guangxi Agri..

[15] Guyonnet V. (2022). Does eggshell color really matter?. WATT Poult. Int..

[16] Huang Q., Qiu N., Ma M.H., Jin Y.G., Yang H., Geng F., Sun S.H. (2012). Estimation of egg freshness using S-ovalbumin as an indicator. Poult. Sci..

[17] Omana D.A., Wu J.P. (2009). A new method of separating ovomucin from egg white. J. Agric. Food Chem..

[18] Kristl M., Jurak S., Brus M., Sem V., Kristl J. (2019). Evaluation of calcium carbonate in eggshells using thermal analysis. J. Therm. Anal. Calorim..

[19] Umbrako I., Petjukevis A., Batjuka A., Harlamora N. (2021). Evaluation of calcium carbonate content in eggshells of avian; turtle; snail; and ostrich using chemical analysis and scanning electron microscopy. Environment, Technologies Resources, Proceedings of the International Scientific and Practical Conference, Rezekne, Latvia, 17–18 June 2021.

[20] Stadelman W.J., Cotterill O.J. (1995). Egg Science and Technology.

[21] You Z.Q., Li B.Y., Jia F., Liu Y., Li X.M. (2020). Study on hen egg quality during storage after transportation. Sci. Technol. Food Ind..

[22] Wang Y.Y., Wang Z.H., Shan Y.Y. (2019). Assessment of the relationship between ovomucin and albumen quality of shell eggs during storage. Poult. Sci..

[23] Abeyrathne E.D.N.S., Huang X., Ahn D.U. (2019). Advances in the Separation of Functional Egg Proteins-Egg White Proteins.

[24] Huang Q., Liu L., Wu Y.Y., Huang X., Wang G.Z., Song H.B., Geng F., Luo P. (2022). Mechanism of differences in characteristics of thick/thin egg whites during storage: Physicochemical, functional and molecular structure characteristics analysis. Food Chem..

[25] Scott T.A., Silversides F.G. (2000). The effect of storage and strain of hen on egg quality. Poult. Sci..

[26] Akyurek H., Okur A.A. (2009). Effect of storage time; temperature and hen age on egg quality in free-range layer hens. J. Anim. Vet. Adv..

[27] Smith M.B. (1964). Studies on ovalbumin I. Denaturation by heat; and the heterogeneity of ovalbumin. Aust. J. Biol. Sci..

[28] Kato A., Ogato S., Matsudomi N., Kobayashi K. (1981). Comparative study of aggregated and disaggregated ovomucin during egg white thinning. J. Agric. Food Chem..

[29] Miller S.M., Kato A., Nakai S. (1982). Sedimentation equilibrium study of the interaction between egg white lysozyme and ovomucin. J. Agric. Food Chem..

[30] Adhikari S., Sharma S.P. (2022). Non-genetic factors influencing internal egg quality traits in chicken: A review. Russ. J. Agric. Socio-Econ. Sci..

[31] Kim C.H., Song J.H., Lee J.C., Lee K.W. (2014). Age-related changes in egg quality of Hy-Line brown hens. Int. J. Poult. Sci..

[32] Nowaczewski S., Lewko L., Kucharczyk M., Stuper-Szablewska K., Rudzińska M., Cegielska-Radziejewska R., Biadala A., Szluc K., Tomczyk T., Kaczmarek S. (2021). Effect of laying hens age and housing system on physicochemical characteristics of eggs. Ann. Anim. Sci..

[33] Biesiasa-Drzazga B., Banaszewska D., Kaim-Mirowski S. (2022). Analysis of selected external and internal characteristics of the eggs of Hy-Line Brown hens in relation to their age. Anim. Sci. Genet..

[34] Moula N., Antoine-Moussiaux N., Decuypere E., Farnir F., Mertens K., De Baerdemaeker J., Leroy P. (2010). Comparative study of egg quality traits in two Belgian local breeds and two commercial lines of chickens. Arch. Geflügelkd..

[35] Sokołowicz Z., Dykiel M., Krawczyk J., Augustyńska-Prejsnar A. (2019). Effect of layer genotype on physical characteristics and nutritive value of organic eggs. CyTA-J. Food..

[36] Lordelo M., Cid J., Cordovil C.M.D.S., Alves S.P., Bessa R.J.B., Carolino I. (2020). A comparison between the quality of eggs from indigenous chicken breeds and that from commercial layers. Poult. Sci..

[37] Hejdysz M., Nowaczewski S., Perz K., Szablewski T., Stuper-Szablewska K., Cegielska-Radziejewska R., Tomczyk L., Przybylska-Balcerek A., Buko M., Kaczmarek S.A. (2023). Influence of the genotype of the hen (*Gallus gallus* domesticus) on main parameters of egg quality, chemical composition of the eggs under uniform environmental conditions. Poult. Sci..

[38] Monira K.N., Salahuddin M., Miah G. (2003). Effect of breed and holding period on egg quality characteristics of chicken. Int. J. Poult. Sci..

[39] Pu J.H., Ge Q.L., Gao Y.S., Zhang B., Xu J.Q. (2010). Study on the regular pattern of quality changes during the egg storage from different chicken breeds. China Poult..

