# Supplementary figures and images for: Physicochemical Index Analyses of the Egg White in Blue-Shelled Eggs and Commercial Brown-Shelled Eggs during Storage
Source: Foods. 2023 Dec 11;12(24):4441. doi: 10.3390/foods12244441 (PMC10742541; doi:10.3390/foods12244441)

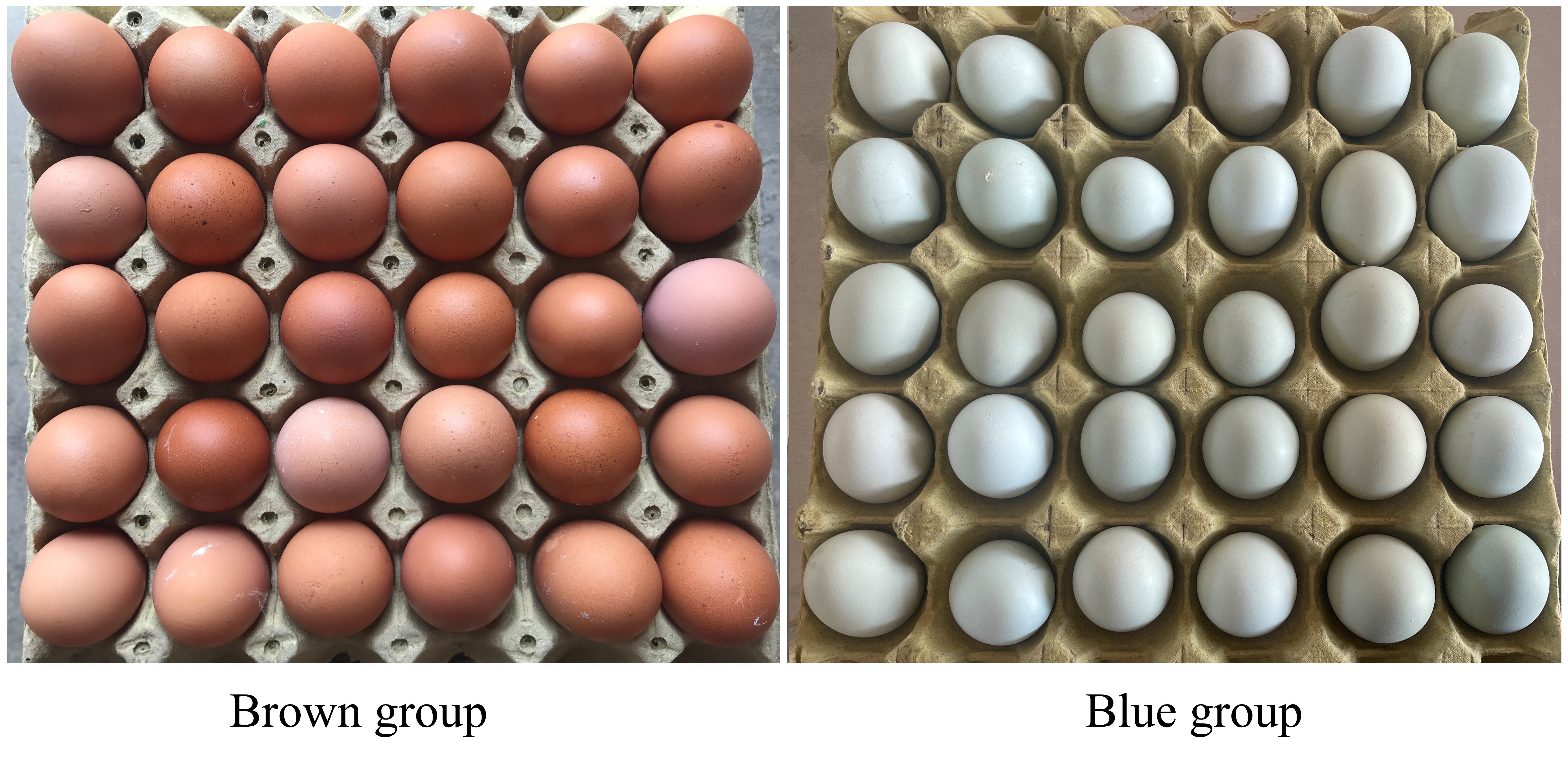

Supplement: Supplementary file 1 [file foods-12-04441-s001.zip › foods-2720255-supplementary.jpg]
